# Predictors of Mortality From a Population-Based Cancer Registry Data in Jos, Nigeria: A Resource-Limited Setting

**DOI:** 10.3389/fmed.2020.00227

**Published:** 2020-06-04

**Authors:** Olugbenga Akindele Silas, Jonah Musa, Tolulope Olumide Afolaranmi, Atiene Solomon Sagay, Charlesnika Tyon Evans, Chad J. Achenbach, Lifang Hou, Robert Leo Murphy

**Affiliations:** ^1^Department of Pathology, University of Jos, Jos, Nigeria; ^2^Feinberg School of Medicine, Northwestern University, Chicago, IL, United States

**Keywords:** cancer registry, human immunodeficiency virus, mortality, cancer, Nigeria

## Abstract

**Background:** It is a well-documented fact that world-wide cancer incidence and mortality remains high in Human Immunodeficiency Virus (HIV) infected population despite potent antiretroviral therapy. With the current capture of HIV status of cancer patients in our cancer registry at Jos Nigeria, this study aims to assess the effect of HIV on cancer mortality outcomes.

**Methodology:** We conducted a 2-year retrospective cohort study of cancer registry data from Jos, north central Nigeria. The cancers were grouped into cervical, breast, liver, hematologic, colonic, AIDS defining, prostate and others in this study. Patients were followed up to determine their patient time contribution from time at initiation of cancer treatment to death or the end of study period. Those lost to follow-up were censored at date of their last known follow-up in clinic.

**Results:** Out of 930 cancer cases evaluated, 52 (5.6%) were HIV positive, 507 (54.5%) were HIV negative and 371 (39.9%) did not know their HIV status. After 525,223 person- days of follow-up, there were 232 deaths leading to a crude mortality rate of 4.3 per 10,000 person-days. Median survival probability for both HIV-infected and HIV uninfected patients were equal (1,013 days). Unadjusted hazard of death was associated with greater age, HR 0.99 (95% CI: 0.98,0.99, *p* = 0.002); hepatitis virus, HR 2.40 (95% CI: 1.69,3.43, *p* = 0.001); liver cancer, HR 2.25 (95% CI:1.11,4.55, *p* = 0.024); prostate cancer, HR 0.17 (95% CI: 0.06,0.393, *p* = 0.001). In an adjusted model, only prostate cancer AHR 0.23 (95% CI: 0.12, 0.42, *p* < 0.001) and liver cancer AHR 2.45 (95% CI: 1.78, 5.51, *p* < 0.001) remained significantly associated with death regardless of HIV status.

**Conclusion:** Having liver cancer increases risk for mortality among our cancer patients. Screening, early detection and treatment are therefore key to improving dismal outcomes.

## Introduction

Globally, of the total 9.6 million cancer deaths worldwide, 50.6% was accounted for by lung, colo-rectum, stomach, liver and breast cancers (1). Out of the 18.1 million world cancer incidence cases reported in 2018, lungs, breast, colorectal, prostate and stomach cancers accounted for 46.1% (1). According to Globocan 2018, there are 115,950 new cancer cases with 70,327 deaths due to cancer in Nigeria (2). Cancers of the breast, cervix uteri, prostate, non-Hodgkin lymphoma, liver, colon, ovary, rectum, leukemia and stomach are reported to be the top 10 commonest cancers in Nigeria in 2018 (2).

The absence of a reliable and quality cancer incidence data in low and middle income countries (LMICs) is well-documented bringing to bear the need for urgent and stringent actions at addressing this (2). Entrenched in the National Cancer Control plan of Nigeria (2018–2022) is the need to improve the existing cancer registration process in Nigeria through adequate funding and increasing the number of staffs of the various cancer registries (3). According to the National Agency for the control of AIDS (NACA) 2018, the national prevalence rate of HIV in Nigeria is 1.4% among adults aged 15–49 years (4). Though this rate has been reducing from the previous estimates of 2.8% the size of Nigeria's population means 1.9 million people were living with HIV in 2018 (4). Several published literature alludes to the fact that there is increased incidence of “HIV-associated cancers” amongst HIV population consisting of both the Acquired Immune Deficiency Syndrome(AIDS)- defining cancers (Kaposi sarcoma, aggressive B-cell non-Hodgkin lymphoma and cervical cancer) and non-AIDS-defining cancers (anal, liver, oral cavity/pharynx, lungs, and Hodgkin lymphoma) (5, 6). Thus, people infected with HIV are 19 times more likely to be diagnosed with anal cancer, 3 times as likely to be diagnosed with liver cancer, 2 times as likely to be diagnosed with lung cancer, about 2 times as likely to be diagnosed with oral cavity/pharynx cancer, and about 8 times more likely to be diagnosed with Hodgkin lymphoma compared with the general population (7). Survival outcomes are worse in HIV- associated than in the non-HIV cancer patients (5–7). The high population of people living with HIV in Jos, Nigeria and its known association with cancers necessitated this study which is to access the effect of HIV on cancer mortality.

## Methods

### Study Setting and Design

We conducted a 2-year retrospective cohort study from January 1, 2016 to March 31, 2018 using data on cancer patients from the population-based Jos cancer registry maintained by the Jos University Teaching Hospital (JUTH) in Jos, Plateau state Nigeria. Plateau state is located in Nigeria's middle belt with an area of 26,899 square kilometers and an estimated population of about 3 million people in 2019 (8). The registry receives cancer data from various health institutions in all the 17 local Government areas of the state. We also included admission and clinic records of these patients. Patients were followed starting at initiation of cancer treatment to occurrence of the primary outcome (death) or the end of study period. Those lost to follow-up were censored at date of their last known follow-up in clinic. We excluded patients who were pregnant and those with incomplete data.

### Data Collection and Measures

Variables collected included HIV sero-status which is usually not part of the evaluated protocol at diagnosis of cancer but information on HIV status of the subject is usually elicited as known or not known and further categorized as positive or negative for those with known HIV sero status from previous HIV screening and confirmation. Cancer topography, hepatitis B and C status, mortality data, demographics (age, sex) and family history of cancer were obtained. The cancers were grouped into cervical, breast, liver, hematologic, colonic, AIDS defining, prostate and others in this study.

### Socio-Demographic Variables Measured

Sex was either male or female; age was analyzed as both continuous and categorized variable; family history of cancer was made into a binary variable as present or absent.

### Clinical Variables Measured

The main exposure variable was HIV sero-status defined by HIV antibody testing. Cancer topography was grouped into cervical, breast, liver, hematologic, colonic, AIDS defining, prostate and others in this study. Histologic tumor type was based on hematoxylin and eosin stain microscopic pathology report. The primary outcome variable was all-cause mortality and was obtained from clinic record sources and the cancer registry for those whose clinic records were absent.

### Statistical Analysis

The analytical focus of this study was to determine all-cause probability of death. The overall mortality rate was estimated using total number of death events and the cumulative person time follow-up from initiation of cancer treatment to death. Differences in proportions and means of observable measures between the HIV-infected vs. HIV-uninfected patients were assessed using Chi-square test for proportions and *t*-test for difference of means. Cox proportional hazard analysis (unadjusted) was done where hazard ratio (HR) and 95% Confidence Intervals (CIs) were used as point and interval estimates of the effects of apriorically selected variables such as age, sex, cancer topography(cervical, breast, colonic, hematological, prostate, AIDS defining, liver and others), HIV infection and Hepatitis infection on all-cause mortality in the cancer patients. Cervical cancer though AIDS defining, was separated from others because of its high prevalence. Adjusted analysis was then conducted where only significant values with a hazard ratio not including 1 were used in the final cox model. All statistical tests were 2-sided with type 1 error set at 0.05 for statistical significance. Sex, HIV infection and Hepatitis infection were all treated as binary variables for determining predictors of death. To estimate the hazard of death following initiation of cancer treatment, we used time from initiation of cancer treatment in days as time covariate and death as failure event. Kaplan-Meier graphs estimating the probability of survival following initiation of cancer treatment by HIV status was plotted. The Log-rank test was used to assess differences in the probability of survival between the HIV positive and HIV negative cancer groups with *p*-value 0.05 signifying significant difference in survival. Statistical analysis was performed with STATA version 11.0 college station, Texas, USA.

## Results

Out of 930 cancer cases evaluated, 52 (5.6%) were HIV positive, 507 (54.5%) were HIV negative and 371 (39.9%) did not know their HIV status. Thus, a total of 559 (60.1%) had their HIV status known either as positive or negative while testing for HIV after cancer diagnosis (Tables 1–3).

**Table 1:** Characteristics of the subjects in the study.

**Characteristics**	**Frequency**	**Percentage (*n* = 930)**
**Age group (years)**		
≤ 40 > 40	282 648	30.3 69.7
**Sex**		
Male Female	494 436	53.1 46.9
**Religion**		
Christianity Islam	792 138	85.2 14.8
**Education status**		
Non formal Formal	157 773	16.9 83.1
**Hepatitis viral status**		
Positive Negative Not known	164 272 494	17.6 29.3 53.1
**HIV status**		
Positive Negative Not known	52 507 371	5.6 54.5 39.9
**Family history**		
Negative Positive	903 27	97.1 2.9
**Outcome**		
Alive Dead	698 232	75.1 24.9
**Topography**		
Hamatological Others^*^ Breast Cervical Colon Liver Prostate AIDS defining+	130 206 181 45 40 150 150 28	14.0 22.2 19.5 4.8 4.3 16.1 16.1 3.0

*+, Non-Hodgkin lymphoma, kaposis sarcoma*.

*, *all other cancers other than hematological, breast, kaposis sarcoma, non-Hodgkin lymphoma, cervical, colon, liver, and prostate cancers*.

**Table 2:** Relationship of the characteristics of the subjects and their HIV Status.

**Characteristics**	**HIV status**				
	**Known (*****n*** **=** **559) Not known (*****n*** **=** **371)**			**χ^2^**	***p***
	**Freq (%)**	**Freq (%)**	**Total**		
**Age group (years)**					
≤ 40 > 40	188 (66.2) 371 (57.3)	94 (33.3) 277 (42.7)	282 648	7.262	0.007
**Sex**					
Male Female	284 (57.8) 275 (63.1)	210 (42.5) 161 (36.9)	494 436	3.011	0.083
**Religion**					
Christianity Islam	478 (60.4) 81 (58.7)	314 (39.6) 57 (41.3)	792 138	0.135	0.714
**Education status**					
Non formal Formal	89 (56.7) 470 (64.1)	68 (43.3) 303 (35.9)	157 773	0.921	0.337
**Hepatitis viral status**					
Known^**^ Not known	416 (95.4) 143 (28.9)	20 (4.6) 351 (71.1)	436 494	426.681	<0.001
**Family history**					
Negative Positive	549 (60.8) 10 (37.0)	354 (39.4) 17 (63.0)	903 27	6.172	0.013
**Outcome**					
Alive Dead	400 (57.3) 159 (68.5)	298 (42.7) 73 (31.5)	698 232	9.155	0.002
**Topography**					
Hamatological Others^*^ Breast Cervical Colon Liver Prostate AIDS defining+	87 (66.9) 121 (58.7) 94 (51.9) 33 (73.3) 25 (62.5) 132 (88.0) 48 (32.0) 19 (67.9)	43 (33.1) 85 (41.3) 87 (48.1) 12 (26.7) 15 (37.5) 18 (12.0) 102 (68.0) 9 (32.1)	130 206 181 45 40 150 150 28	109.892	<0.001

*+. kaposis sarcoma, non-Hodgkin lymphoma*.

*, *all other cancers other than hematological, breast, cervical, colon, AIDS defining, liver, and prostate cancers*.

**, *Either positive or negative for hepatitis B or and C*.

**Table 3:** A bivariate analysis of demographic/clinical characteristics data of cancer patients documented at the cancer registry from 01/01/2016 to 03/30/2018 by HIV status.

	**HIV positive *n* (%)**	**HIV negative *n* (%)**	**Unknown**	**Overall**	***P*-value**
Number	52 (5.60)	507 (54.5)	371 (39.9%)	930 (100.00)	
**Sex**					
Male Female	20 (7.04) 32 (11.64)	264 (92.96) 243 (88.36)			0.062
**Age (yrs)**					
≤ 40 > 40	20 (10.47) 32 (8.70)	171 (89.53) 336 (91.30)			0.540
Age	median(IQR) yrs	50 (38, 63)			
**Religion**					
Christianity Islam	47 (9.85) 5 (6.17)	430 (90.15) 76 (93.87)			0.408
**Hepatitis**					
Negative Positive	16 (5.97) 7 (4.73)	252 (94.03) 41 (95.27)			0.661
**Family history**					
Negative Positive	40 (13.51) 1 (10.00)	256 (86.49) 9 (90.00)			1.000
**Outcome**					
Alive Dead	45 (11.25) 7 (4.40)	355 (88.75) 152 (95.60)			0.010
**Topography**					
Hematological Others* Breast Cervical Colon Liver Prostate AIDS defining	10 (11.49) 9 (7.4) 9 ((9.57) 25 (75.76) 1 (04.00) 3 (2.27) 5 (10.42) 12 (63.20)	77 (88.51) 112 (92,60) 85 (90.43) 8 (24.24) 24 (96.00) 129 (97.73) 43 (89.58) 7 (36.80)			<0.001

Yrs = years; others* = all other cancers other than hematological, breast, cervical, colon, AIDS defining, liver and prostate cancers; Hepatitis = hepatitis B or and C virus positive.

**Figure 1:**
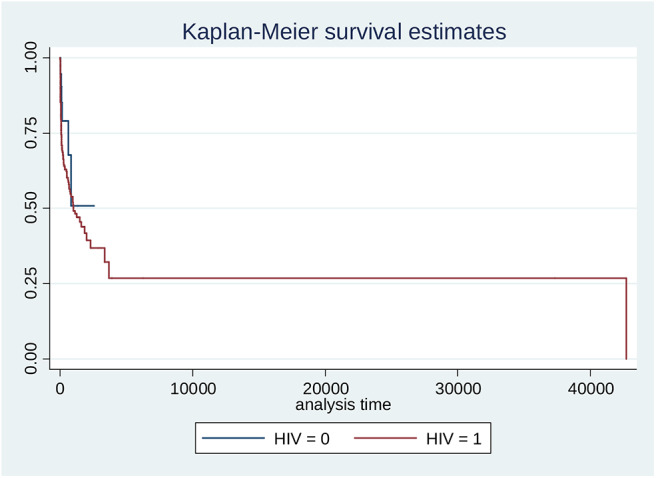
Kaplan Meier estimation of survival stratified by HIV status (HIV positive = black/0; HIV negative = red/1). Log-rank test for equality of survivor functions: chi2 = 3.23; Pr>chi2 = 0.0722.

The commonest cancers irrespective of HIV status were breast cancer 181 (19.5%), liver cancer 150 (16.1%), prostate cancer 150 (16.1%), and cervical cancer 45 (4.8%). Proportions for other cancers known to be associated with HPV, EBV, and HHV8 were: oropharngeal cancers 15 (1.6%), Non-Hodgkin Lymphoma 10 (1.1%), and Karposis sarcoma 9 (0.9%), respectively, out of which over 70% occurred in HIV positive patients (Tables 1–3). Out of a total of 930 cancer cases evaluated 648 (69.7%) were of age group > 40 years and 494 (53.1%) were males (Tables 3 and 4). Median age of the participants was 50, 25 and 75% interquartile range is 38 and 63. After 525,223 person- days of follow-up, there were 232 deaths leading to a crude mortality rate of 4.3 per 10,000 person-days. Median survival probability for both HIV-infected and HIV uninfected patients were equal (1,013 days) (Figure 1). Unadjusted hazard of death was associated with greater age, HR 0.99 (95% CI: 0.98,0.99, *p* = 0.002); hepatitis virus, HR 2.40 (95% CI: 1.69,3.43, *p* = 0.001); liver cancer, HR 2.25 (95% CI: 1.11,4.55, *p* = 0.024); prostate cancer, HR 0.17 (95% CI: 0.06,0.39, *p* = 0.001) (Table 5). In an adjusted model, only liver cancer AHR 2.45 (95% CI: 1.17, 5.51, *p* < 0.001) and prostate cancer AHR 0.23 (95% CI: 0.12, 0.42, *p* < 0.001) using others as reference significantly predicted death regardless of HIV status (Table 5). The log-rank test for Kaplan-Meier graph was not significantly different between those with and without HIV infection (*P* = 0.072).

**Table 4:** Summary of characteristics of the study subjects by Cancer Type.

**Characteristics *n* = 930**	**Topography**							
	**Haem Freq (%)**	**Breast Freq (%)**	**Cervical Freq (%)**	**Colon Freq (%)**	**Liver Freq (%)**	**Prostate Freq (%)**	**AIDS defining + Freq (%)**	Others^*^ Freq (%)
**Age group (years)**								
≤ 40 (*n* = 282) > 40 (*n* = 648)	58 (20.6) 72 (11.1)	52 (18.4) 129 (19.9)	12 (4.3) 33 (5.1)	12 (4.3) 28 (4.3)	44 (15.6) 106 (16.4)	0 (0.0) 150 (23.1)	7 (2.5) 21 (3.2)	97 (34.4) 109 (16.8)
**Sex**								
Male (*n* = 494) Female (*n* = 436)	78 (15.8) 52 (11.9)	7 (1.4) 174(39.9)	0 (0.0) 45 (10.3)	25 (5.1) 15 (3.4)	116 (23.5) 34 (7.8)	150 (30.4) 0 (0.0)	20 (4.0) 8 (1.8)	98 (19.8) 108 (24.8)
**Religion**								
Christian (*n* = 792) Islam (*n* = 138)	110 (14.0) 20 (14.5)	155 (19.7) 26 (18.8)	37 (4.7) 8 (5.8)	31 (3.9) 7 (5.1)	140 (17.8) 10 (7.2)	129 (16.4) 19 (13.8)	21 (27.0) 7 (5.1)	165 (20.8) 41 (29.7)
**Education status**								
Non formal (*n* = 157) Formal (*n* = 773)	17 (10.8) 113 (14.6)	21 (13.4) 160 (20.7)	10 (6.4) 35 (5.5)	6 (3.8) 34 (4.4)	26 (16.6) 124 (16.0)	35 (22.3) 115 (14.9)	1 (0.6) 27 (3.4)	41 (26.1) 165 (21.3)
**Hepatitis viral status**								
Known^**^ (*n* = 436) Not known (*n* = 494)	64 (14.7) 66 (13.4)	67 (15.4) 114 (23.1)	21 (4.8) 24 (4.9)	18 (4.1) 22 (4.5)	142 (32.6) 8 (1.6)	34 (7.8) 116 (23.5)	7 (1.6) 21 (4.3)	83 (19.0) 123 (24.9)
**Family history**								
Positive (*n* = 27) Negative (*n* = 903)	1 (3.7) 129 (14.3)	15 (55.6) 166 (18.4)	2 (7.4) 43 (4.8)	1 (3.7) 39 (4.3)	2 (7.4) 43 (16.4)	2 (7.4) 148(16.4)	0 (0.0) 28 (3.1)	4 (14.8) 202 (22.4)
**Outcome**								
Alive (*n* = 698) Dead (*n* = 232)	103 (14.8) 27 (11.6)	142 (20.3) 39 (16.8)	34 (4.9) 11 (4.7)	30 (4.3) 10 (4.3)	91 (13.0) 59 (25.4)	137 (19.6) 13 (5.6)	19 (2.7) 9 (3.9)	142 (20.3) 64 (27.6)
**HIV status** ***n*** **=** **559**								
Positive (*n* =52) Negative (507)	10 (19.2) 77 (15.2)	9 (17.3) 85 (16.8)	8 (15.4) 25 (4.4)	1 (1.9) 24 (4.7)	3 (5.8) 129 (25.4)	5 (9.6) 43 (8.5)	7 (13.5) 12 (2.4)	9 (17.3) 112 (22.1)

*, *all other cancers other than hematological, breast, kaposis sarcoma, non-Hodgkin lymphoma, cervical, colon, liver and prostate cancers*.

*+, Non-Hodgkin lymphoma, kaposis sarcoma*.

** *Either positive or negative for hepatitis B or and C*.

**Table 5:** Unadjusted and Adjusted analysis of characteristics of cancer patients using cox- proportional hazards.

**Variable**	**Unadjusted HR** **(95% CI)**	***p*-value**	**Adjusted HR** **(95% CI)**	***p*-value**
Age	0.99 (0.98,0.99)	0.002	0.998 (0.99,1.01)	0.549
**Hepatitis virus status**				
Known	2.40 (1.69,3.43)	0.001	1.274 (0.952,1.704)	0.103
Unknown	1	–	–	–
Topography^*^				
AIDS defining	1	–	–	–
Liver	2.25 (1.110, 4.550)	0.024	2.45 (1.78,5.51)	<0.001
Prostate	0.17 (0.06,0.393)	0.001	0.23 (0.12,0.42)	<0.001

*HR – Hazard ratio; CI, confidence interval; Hepatitis virus (known = hepatitis B or and C virus positive)*.

*, *Reference category for the topography of cancer in the adjusted model is any cancer type other than liver and prostate*.

### Adjusted Cox Model

In an adjusted model, prostate cancer AHR 0.23 (0.12, 0.42, *p* < 0.001) and liver cancer AHR 2.45 (95% CI: 1.78, 5.51, *p* < 0.001) using others as reference significantly predicted death positively and negatively, respectively, regardless of HIV status (Table 5).

### Kaplan-Meier Survival Graph for Separate HIV Groups (Figure 1)

The log-rank test for Kaplan-Meier graph was not significantly different between those with and without HIV infection (*P* = 0.072). Median survival probability for HIV-infected and HIV-uninfected group was 1,013 days each (Figure 1).

$$\begin{array}{l} 0=\text{HIV positive }1\;=\text{ HIV negative} \end{array}$$

## Discussion

In this 2-year retrospective cohort study, the significant proportion of cancer patients observed with unknown HIV status 371 (35.6%) could be attributed to the fact that testing for HIV status of cancer patients was not considered as a standard of care in the management of cancer patients in the various health institutions in our setting. It is now being advocated that healthcare providers should request for HIV testing for all cancer patients being managed as HIV has been well-documented to have a significant impact on ultimate survival outcomes of cancer patients (8–19). All those cases identified in this study with known HIV status had indicated their status in the course of cancer care and recorded in the registry as such.

Probability of survival among the HIV positive and HIV negative cohorts was not significantly different (Pr = 0.072). This is contrary to most reports on mortality after cancer diagnosis which showed higher mortality among HIV seropositive cancer patients than their HIV sero-negative counterparts (16–20). In a study on the contribution of HIV infection to mortality among cancer patients in Uganda, a more than 2-fold increased risk of death among the HIV-infected cancer patients compared to HIV- uninfected cancer patients was reported. Though in their study only five major cancers were considered and patients were at least 18 years of age (21). Most of the patients in this study were of age group > 40 years consistent with the age group with frequent age associated cancers as reported in other similar studies (22–26). Our relatively equal probability of survival between HIV positive and HIV negative cohorts could be attributed to the possible diffusing effect of unknown HIV status in this study as some of them could have been sero- positive. Also is the relatively low sample size of those with known HIV positive status.

Our study showed that patients with liver cancer were about three times more likely to die than those with other cancers regardless of HIV status. This might be due to inadequate screening facilities and challenges faced with early diagnosis of liver pathology especially in low and middle income countries (LMICs). However, surgical interventions having the possibility of prolonging lives at early stage diagnosis are also not readily available in this setting. Most patients with liver cancer thus present at advanced stages due to illiteracy, poverty and reliance on the readily available and cheap local traditional remedies (27, 28). There is also increased prevalence of risk factors of liver cancer in our communities such as aflatoxin, hepatitis B and C (27, 28). Probability of death due to prostate cancer however was low as fatality due to prostate cancer is generally known to be relatively rare.

Limitations of this study includes use of secondary observational data, small size of the HIV cohort, lack of important information that could impact survival such as actual mode of cancer treatment, cancer stage and grade and the unavailability of important HIV clinical data such as CD4 count and viral load.

In spite these limitations, our study has several strengths, to our knowledge this is the first study to determine effect of HIV on cancer mortality outcomes in our sub-region. This study has also revealed the fact that most of our patients are not aware of their HIV status. This underscores the need to increase HIV screening uptake in our population and the need to institute HIV screening as part of standard of care in cancer patients. Results of this study could serve as baseline for future large prospective studies that could be replicated in other regions of the country for generalizability.

## Conclusion

Cancers have remained a vital cause of mortality in our setting with high HIV population thus necessitating screening and early detection to improve outcomes. Having liver cancer increases risk for mortality among our cancer patients. Hence, screening for premalignant lesions, early detection and treatment of cancers generally are therefore key to improving dismal outcomes. However, the contributions of HIV infection to cancer mortality still need to be further explored particularly if HIV testing is institutionalized as part of the standard of care for cancer patients and by the linkage of necessary HIV clinic data to the cancer registry data.

## Data Availability Statement

The datasets generated for this study are available on request to the corresponding author.

## Ethics Statement

The studies involving human participants were reviewed and approved by Jos University Teaching Hospital's Ethical committee. Written informed consent from the participants' legal guardian/next of kin was not required to participate in this study in accordance with the national legislation and the institutional requirements.

## Author Contributions

OS: conceptualized the study. JM, TA, CA, and LH: made technical inputs in the conceptualization and design of the study. OS, JM, CE, AS, and LH: produced the first draft of the manuscript. AS, RM, LH, and CE: contributed in further interpretation of findings and editing of the final draft of the manuscript. All co-authors contributed in revising the manuscript and approved the final version for submission.

## Conflict of Interest

The authors declare that the research was conducted in the absence of any commercial or financial relationships that could be construed as a potential conflict of interest.
